# Practical Departments

**Published:** 1906-02-24

**Authors:** 


### PRACTICAL DEPARTMENTS.

### THE BASTIAN MERCURY VAPOUR ARC-LAMP.

This is an electric lamp in which the light is given out by the glowing vapour of mercury, and it is very rich in the violet rays of the spectrum, while the red rays are stated to be quite absent. Paper and fabrics that look red in sunlight look black by the light of the mercury-vapour arc. In the Bastian lamp there is a small tube having bulbs containing mercury at each end, with platinum electrodes sealed into the bulbs. When the lamp is not burning, the mercury runs along the tube, making electrical connection between the bulbs. An electro-magnet tilts the tube when the current is switched on, breaking the connection, and an arc is formed which fills the whole of the tube, giving out the light mentioned. The tube is made in different forms, a favourite one being a ring held under a reflector. It is also made to be started by hand, by tilting it. Being so rich in the violet rays, it may be of service in cases where the Finsen light has been used. The spectrum is stated to include only the violet, blue, green, yellow, and orange rays. The lamp is sold by Messrs. Rumney and Rumney of 39 Victoria Street, London, S.W.

### MESSRS. THOMAS AND TAYLOR'S LAUNDRY MACHINERY.

Messrs. Thomas and Taylor, whose works are at Stockport, and who have a depot at 99 Fonthill Road, Finsbury Park, London, N., are makers of laundry machinery of all kinds, for which, and for dairy machinery which they also manufacture, they have been awarded upwards of forty prize medals at various exhibitions. They make every kind of washing, wringing, and drying apparatus, among their specialities being a soap and soda tank attached to the washing machine itself, in which the soap and soda are prepared, thus avoiding the necessity of carrying them about the laundry in a boiling state; and a special arrangement of the inner cylinder of the washing machine. The washing cylinder is fitted with four stout inner ribs, as in other apparatus, which perform the office of scrubbing. In Messrs. Thomas and Taylor's machine the ribs are hollow, water being allowed to pass into them, and from there being sprayed on to the clothes, through holes in the ribs; the lid of the washing machine, also, is securely fastened with one turn. They make a pressure disinfecting washing machine, arranged so that the soap and soda can be put in whilst the machine is at work, and a special form of clothes boiler, consisting of an iron or copper boiler of the ordinary pattern with a perforated case of similar shape inside. When the clothes have been boiled sufficiently, the inner case is lifted out by a rope attached to a small winch, and can then be tipped by one woman. Besides the usual drying closets, with horses to pull out, Messrs. Thomas and Taylor make portable fireproof hot air drying and disinfecting closets, with an ironers' stove attached. They are built of iron throughout, and contain two horses for drawing out, the heat for drying being supplied by the stove that is also used for heating the irons. The apparatus is very strongly built, and in such a manner that it can be taken to pieces, packed, and sent anywhere for temporary use. A flue is, of course, necessary, leading into the open air, to carry off the products of combustion from the fire. The heating stove is also made separately for irons. The firm make all the usual lines of callenders, goffering, and other finishing machines, in which they have their own specialities. They supply engines, boilers, and other accessories for engineering work generally.

